# Young Shrubs: Promotion of Early Career Scholars in MIDUS

**DOI:** 10.1093/geroni/igaf122.1537

**Published:** 2025-12-31

**Authors:** Daniel Mroczek

**Affiliations:** Northwestern University, Evanston, Illinois, United States

## Abstract

From its earliest days, MIDUS leadership involved and included early career scholars. Access to MIDUS data and networking with senior members promoted the careers of those first generation MIDUS associates in the 1990s. This role modeling has passed down to later generations whereby that first cohort of beneficiaries has mentored later generations in the early 2000s, 2010, and now the 2020s. Second and Third generation MIDUS mentees are now active. This international system of mentoring is exemplified by recent articles that include scholars who were involved at the MIDUS inception as well as those who are much more recent PhDs (2021 or later). Examples of work that not only makes use of three waves of MIDUS data but includes MIDUS first generation scholars as well as much later generations of scholars will be highlighted. The success of long-term longitudinal studies like MIDUS are dependent on such intergenerational connections.

